# Statistical dictionaries for hypothetical in silico model of the early-stage intermediate in protein folding

**DOI:** 10.1007/s10822-015-9839-2

**Published:** 2015-03-26

**Authors:** Barbara Kalinowska, Piotr Fabian, Katarzyna Stąpor, Irena Roterman

**Affiliations:** 1 Department of Bioinformatics and Telemedicine, Jagiellonian University Medical College, Lazarza 16, Krakow, Poland; 2 Faculty of Physics, Astronomy and Applied Computer Science, Jagiellonian University, Reymonta 4, Krakow, Poland; 3 Institute of Computer Science, Silesan Technical University, Akademicka 16, 44-100 Gliwice, Poland

**Keywords:** Early stage folding, Folding intermediate, Structure predictability, Folding process, Folding simulation

## Abstract

The polypeptide chain folding process appears to be a multi-stage phenomenon. The scientific community has recently devoted much attention to early stages of this process, with numerous attempts at simulating them—either experimentally or in silico. This paper presents a comparative analysis of the predicted and observed results of folding simulations. The proposed technique, based on statistical dictionaries, yields a global accuracy of 57 %—a marked improvement over older approaches (with an accuracy of approximately 46 %).

## Introduction

Ab initio protein structure prediction methods (new fold, Boltzmann-based) [1] strongly depend on initial structures. Optimization algorithms tend to produce conformations which either match or closely approach local minima instead of the protein’s native form. Some progress in this regard can be observed by tracking the outcome of the CASP competition (http://www.predictioncenter.org). Experimental analysis indicates that protein folding involves multiple stages [2–8] and this observation is further reinforced by in silico models [9, 10]. The analysis presented in this work assumes a two-stage process [11–14]. We will focus on the so-called Early Stage (ES) intermediate whose structure can be derived on the basis of a limited conformational subspace, restricting the allowed set of (*φ*, *ψ*) angle pairs to an elliptical path on the Ramachandran plot. The rationale behind this restriction is extensively discussed in [15–22] and has been stipulated for many years [23].

### Early stage model (ES)

**Fig. 1:**
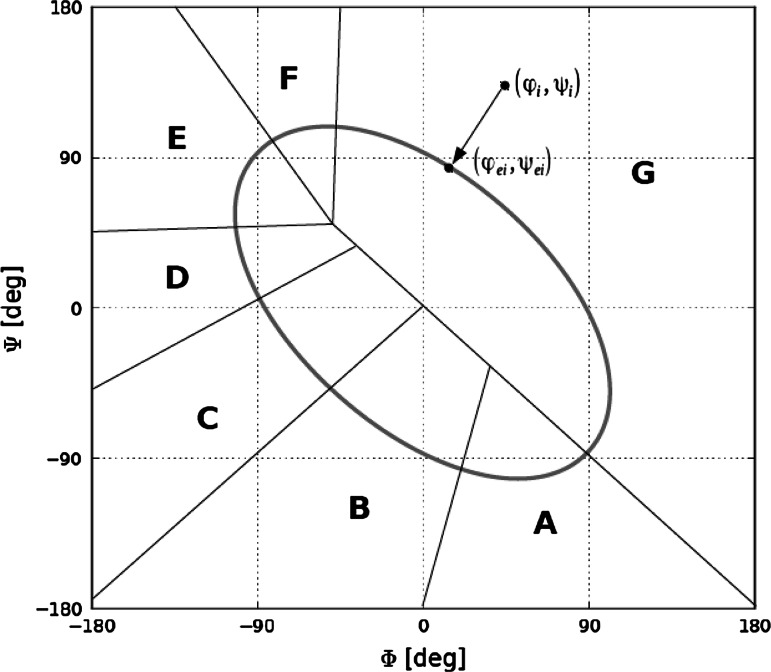
Conformational subspace represented by an elliptical path on the Ramachandran plot (*gray line*), with areas corresponding to local probability distribution maxima of (*φ*_*e*_, *ψ*_*e*_) angle pairs obtained through minimum-distance projections (*black lines*). The *black arrow* depicts a sample projection (*φ*_*i*_, *ψ*_*i*_) → (*φ*_*ei*_, *ψ*_*ei*_)

The ES model assumes that the initial conformation of the polypeptide chain can be predicted on the basis of its backbone, neglecting side chain contributions. In our model the ES intermediate is expected to conform to the previously mentioned limited conformational subspace [12, 15, 16]. This subspace is represented by an elliptical path which traverses areas corresponding to well defined secondary structural motifs on the Ramachandran plot. Its shape and placement follow from analysis of the chain’s backbone structure, expressed using pairs of V-angles, i.e. angles between planes corresponding to two adjacent peptide bonds. This second-order function delineates a path along which the curvature radius matches observed values (Fig. 1).

**Fig. 2:**
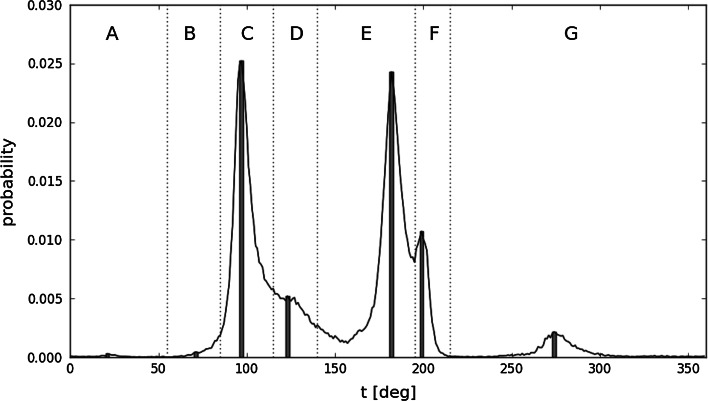
Probability distribution profiles for (*φ*_*e*_, *ψ*_*e*_) values for histidine, along with structural codes corresponding to individual maxima. The *t* parameter traverses the elliptical path starting with one of its poles which is located in the *bottom right-hand* corner of the Ramachandran plot. *Bars* represent the specific placement of all seven probability maxima which are used to express the early stage intermediate structure

If each observed pair of (*φ*, *ψ*) angles is projected onto the limited subspace using the minimum distance criterion, the distribution of the resulting pairs (*φ*_*e*_, *ψ*_*e*_) can be shown to exhibit seven distinct maxima (Fig. 2). The areas corresponding to each local maximum can be translated into a structural code, resulting in a structural alphabet which consists of seven letters (A–G). This alphabet enables us to express the predicted structure of the ES intermediate with the precision of limited conformational sub-space.

### ES structure prediction

Once the structure of the polypeptide chain (as given by PDB) is denoted using the structural codes discussed above, it becomes possible to study the relation between residue sequences and structural codes. This relation can be expressed as a contingency table in which each sequence of amino acids corresponds to a given code with specific probability. Contingency tables can be used to predict the structure of input sequences. While constructing our structural alphabet we have applied the greatest probability criterion and selected tetrapeptide fragments as the basis of our contingency tables.

As already indicated, the ES intermediate structure can be predicted to within the nearest maximum of the limited conformational subspace. Further analysis based on information theory principles indicates that the quantity of information required to make this prediction corresponds closely to the quantity of information which is present in the polypeptide chain itself [16]. The accuracy of structural predictions based on tetrapeptide fragments and contingency tables has been discussed in [24]. In this paper we present a different code selection method, based on statistical dictionaries which permit us to take into account longer input sequences.

### Statistical dictionaries

The newly implemented early-stage secondary structure prediction method is based on statistical dictionaries: we have assembled a dictionary of primary substrings and their corresponding secondary structures. In general, dictionary methods use a large set of items—words, translations, sequences of symbols etc. These methods are applied in many domains: text translation (the dictionary contains a number of phrases with the corresponding translations), speech synthesis, cryptography, etc. Dictionary methods depend on a large set of previously solved problems in order to find a solution to the problem at hand. Even if a direct solution is not present in the dictionary, the solver algorithm may find similar problems and use their solutions to generate a suitable answer.

The presented method is based on the assumption that a sufficiently long substring of the primary structure always leads to the same secondary structure subsequence. The method consists of two stages: dictionary construction stage and prediction stage.

Comparing the presented technique with earlier approaches based on analysis of tetrapeptide fragments indicates that using statistical dictionaries produces a marked increase of accuracy (from 46 to 57 %), rendering our new method superior.

## Materials and methods

### Databases

The dictionary is built on the basis of selected proteins from the PDB database. A nonredundant protein database was generated using the BlustClust tool (http://www.ncbi.nlm.nih.gov/Web/Newsltr/Spring04/blastlab.html). Following elimination of proteins whose degree of sequential similarity was greater than 95 % the database numbered 24820 proteins. The training set consisted of 24426 protein chains while the testing set consisted of 246 protein chains, selected to be dissimilar to chains in the training set. This is essential to ensure, that the prediction stage does not use information about chains from the testing set. Residues involved in interactions with external molecules were identified by measuring the distance between the external molecule and the protein under analysis [a cutoff distance of 2.9 Å was applied, in line with PDBSum standards (http://www.ebi.ac.uk/pdbsum)].

### Statistical dictionaries

Each dictionary contains records composed of two elements: the primary subsequence and corresponding secondary structure for the middle element of the subsequence. Substrings are generated from the training set using a sliding window. Each chain of length *n* generates *n* pairs (substring, secondary structure class). For a given length *l* of the window, [*l*/2] additional neutral ‘X’ symbols are added at the beginning and end of the chain. The sliding window is then moved from left to right, generating pairs. The secondary structure class applies to the middle element in the window. Our implementation collected substrings up to 13 elements long. The dictionary uses a family of hash functions [25] to place all strings in a number of hash tables. Each hash table creates one subdictionary *D*_*i*_, *i* = 1, 3, 5, …, *l*_*max*_. Subdictionary *D*_*i*_ contains strings of length *i*. Each record placed in a dictionary is composed of two elements: the primary string and a set of seven counters counting the occurrences of seven possible structural code classes (A, B, …, G) for the middle element of the primary string.

The prediction algorithm uses information from the dictionary built in the first stage. Each position of input string *p*_*s*_ is analyzed. For each position, subdictionaries *D*_*i*_, *i* = 13, 11, …, *1* are used to match a substring extracted from *p*_*s*_, from position *p*_*s*_[*k* − 2*i*] to *p*_*s*_[*k* + 2*i*]. If a match is found, the corresponding best secondary structure class is retrieved from the dictionary. If an exact match is not found, another try is made to find an approximate match with one non-matching position. If not successful, a smaller value of *i* is taken. The last subdictionary, *D*_*1*_, contains all twenty possible elements so this algorithm always finds a match. Sequence *p*_*s*_ is additionally padded with a sequence of [*i*/2] ‘X’ elements at the beginning and end, which is not shown in the code.

### Evaluation measures for prediction of the 7-class structural alphabet

The evaluation formula is very simple and similar to the Q3 measure. For a given amino acid chain of length n, the observed structural code is denoted as *S*_*ob*s_[1…*n*], and the predicted structural code as *S*_*pred*_[1…*n*]. The accuracy for this amino acid is computed as *m*/*n*, where *m* is the number of indexes *i*, for which *S*_*obs*_[*i*] = *S*_*pred*_[*i*] and *n* is the length of the chain. Accuracies for all 7 classes (A–G) of the structural alphabet have also been computed in a similar way. For each class only positions with *S*_*obs*_[*i*] equal to this class have been taken into account. If there were no elements of this class in the secondary structure, the accuracy for this class was assumed to be 0 % (which may be a bit misleading). The total accuracy for the whole testing set is defined as the arithmetic mean of accuracies for all chains. Total accuracies for 7 classes of the structural alphabet are computed analogously.

### Comparative analysis

Predicted structural codes were compared with secondary structures determined by the DSSP algorithm for structures deposited in PDB [26, 27]. The secondary structures were obtained from the online DSSP database (http://www.cmbi.ru.nl/dssp.html). Additionally, the prediction results were collated with prediction of secondary structures obtained by the SPINE X method [28, 29] for the identical testing set of protein chains. The method distinguishes three secondary structure classes—helical (H), extended (E) and coils (C). In order to draw a comparison, such three groups of structures were created also for ES structural codes and DSSP structures. DSSP structures were grouped as follows—helical structures contain H (α-helix), G (helix-3) and I (helix-4), extended—B (β bridge) and E (strand), coils—T (turn), S (bend) and not classified. The same division was used by authors of SPINE X for evaluating predictions. The ES structural codes can be easily assigned to helical (C) and extended (E and F) structures. The four other codes create the third group but they cannot be identified with turns, bends and coils unambiguously.

## Results

Results summarized in Table 1 present the overall accuracy of the structural code identification method discussed above. The aggregate value of 56.67 % compares favorably to results obtained using contingency tables which assign structural codes to tetrapeptides. Table 1 also shows the prediction accuracy for residue sets obtained by eliminating residues involved in external interactions (with ligands, other proteins or DNA/RNA chains). The differences between all four groups of results are negligible—the statistical dictionary method does not seem to favour non-interacting residues, while the contingency table method is substantially affected by eliminating residues engaged in ligand interaction as shown in [24]. In contrast, elimination of residues which interact with proteins and DNA/RNA does not alter the accuracy of predictions and both methods are quite similar in this scope. Results obtained using the maximum probability criterion are on the order of 46 % and seem affected by the status of each residue (i.e. whether it is involved in external interactions). As shown, this correlation is strongest for residues which bind external ligands and other proteins, whereas interaction with DNA/RNA chains has a limited effect on prediction accuracy. The proposed method does not seem affected by such perturbations—whether due to methodological differences or to the relatively limited representation of interacting residues in the study set. The physical model assumes that the presence of external factors (such as ligands) may affect the local conformation of peptide bonds. Due to its highly specific nature of such distortions we should not expect the resulting conformation to match the “standard” structural form for a given sequence.

**Table 1:** Structural code prediction accuracy (percentage values) for the full set of amino acids and for partial sets obtained by eliminating residues which interact with ligands, other proteins and DNA/RNA. The final row contains values obtained using the contingency table method [24]

Prediction accuracy (%)				
Structural code	Complete set	Amino acids excluded engaged in interaction with		
Total		Ligand	Protein	DNA/RNA
A	18.83	18.71	18.69	18.83
B	9.40	9.50	9.70	9.40
C	72.30	72.47	72.25	72.29
D	27.62	27.29	27.67	27.64
E	54.37	54.24	53.60	54.37
F	36.53	36.57	36.67	36.53
G	44.81	44.97	44.83	44.82
	56.67	56.77	56.69	56.67
Previous results	45.77	45.93	45.92	45.75

The improved accuracy of the statistical dictionary method (which takes into account fragments consisting of 1–13 amino acids) indicates that tetrapeptides are not sufficient for predicting the structure of the resulting chain. Restricting analysis to such short fragments effectively eliminates all nonstandard conformations, while taking into account longer chains may result in (correct) selection of structural forms which occur with lower probability.

### Prediction accuracy for individual amino acids

**Fig. 3:**
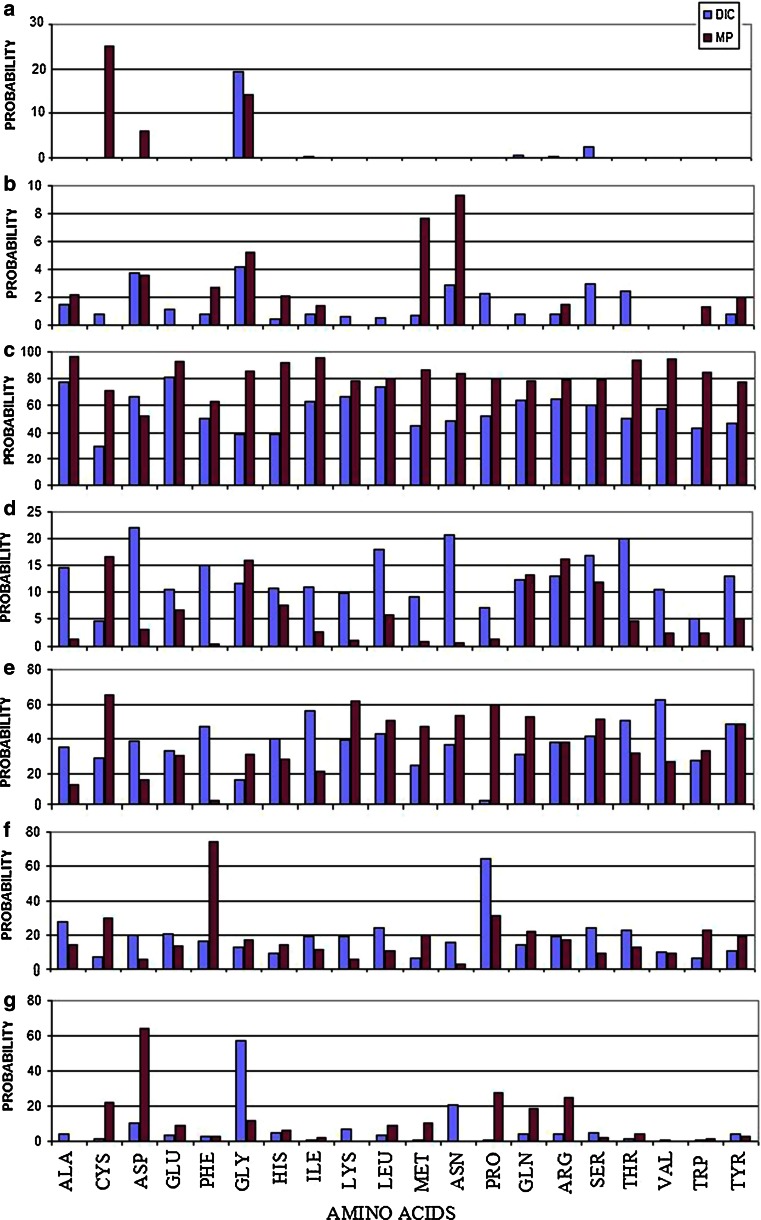
Comparison of prediction accuracy between statistical dictionaries method (DIC) and contingency table approach (based on the Maximum Probability in contingency table—MP) for amino acid residues and individual structural codes. The list of residues is given on the *bottom line*. The zones on Ramachandran map is represented according to symbols **a**–**g**. The **c**—represents the helical area, **e** and **f** the β-structural forms and **g**—left helical area. The codes **a**, **b** and **d** traditionally are treated as Random Coil

Table 2 presents the prediction accuracy for individual amino acids. The presented values (obtained using the statistical dictionary method) hint at specific correlations (Fig. 3).

**Table 2:** Structural code prediction using new method (top row) and the method described in [24] (bottom row)

	Total	A	B	C	D	E	F	G
ALA	60.05	0.00	1.45	77.92	14.44	35.34	27.33	4.47
		0.00	2.20	96.04	1.42	13.38	14.37	0.0
CYS	13.39	0.00	0.81	29.62	4.55	28.87	6.81	1.22
		25.0	0.0	71.37	16.48	65.07	30.0	21.87
ASP	49.17	0.00	3.75	66.39	22.06	38.73	19.85	10.16
		5.87	3.60	52.68	2.96	16.35	5.97	64.34
GLU	62.50	0.00	1.12	81.05	10.43	33.2	20.55	3.65
		0.0	0.0	93.22	6.67	30.32	13.79	8.86
PHE	52.00	0.00	0.81	50.42	14.96	47.19	16.09	3.04
		0.0	2.67	63.04	0.55	2.04	74.54	3.03
GLY	44.00	19.34	4.15	38.98	11.57	16.16	12.47	57.41
		14.28	5.20	85.60	15.84	30.66	16.79	11.7
HIS	36.16	0.00	0.41	38.92	10.76	39.79	9.28	5.01
		0.0	2.08	92.03	7.43	27.98	13.99	6.12
ILE	58.55	0.40	0.81	62.90	10.97	55.86	19.35	0.40
		0.0	1.35	95.69	2.49	21.01	10.99	1.94
LYS	52.81	0.00	0.61	67.02	9.75	39.64	19.30	6.57
		0.0	0.0	78.04	1.06	61.75	5.62	0.0
LEU	58.77	0.00	0.51	73.62	17.88	42.88	24.33	3.25
		0.0	0.0	79.80	5.69	50.84	10.57	8.70
MET	22.21	0.00	0.68	45.02	9.01	24.73	6.23	0.81
		0.0	7.69	86.44	0.92	46.79	9.85	10.34
ASN	45.44	0.00	2.84	48.95	20.69	36.49	15.55	20.49
		0.0	9.33	83.76	0.65	53.19	2.68	0.0
PRO	59.66	0.00	2.23	52.46	7.08	1.83	64.67	0.41
		NA	0.0	80.19	1.37	59.75	30.93	27.78
GLN	51.47	0.41	0.81	63.67	12.33	30.57	14.08	4.27
		NA	0.0	78.35	13.19	52.68	21.6	18.64
ARG	53.00	0.40	0.81	64.86	12.90	38.24	18.78	4.27
		NA	1.47	79.71	16.23	37.86	17.05	24.91
SER	45.61	2.44	2.98	60.25	16.92	41.07	23.94	4.53
		NA	0.0	79.43	11.90	51.54	9.50	2.22
THR	47.26	0.00	2.44	50.06	19.96	50.35	22.70	1.22
		NA	0.0	93.52	4.56	31.53	12.63	4.20
VAL	60.66	0.00	0.00	57.58	10.56	62.48	9.85	0.81
		NA	0.0	94.91	2.21	26.74	8.95	0.0
TRP	25.73	0.00	0.00	43.32	4.94	27.68	6.37	0.41
		NA	1.33	85.13	2.24	32.98	22.4	1.22
TYR	41.26	0.00	0.81	46.4	12.9	48.24	10.51	3.86
		NA	2.0	77.55	5.07	48.58	18.88	3.03

Major differences can be observed for C-type structures (clockwise α-helix) and for cysteine. The presented method is less apt to propose α-helical forms for all residues except aspartic acid. D- and F-type structures are predicted with greater accuracy for most residues. Code D represents transitional structures which form the bridge between the α-helix and β-twist areas on the Ramachandran plot. Likewise, code F is adjacent to the β-twist area, aggregating forms with low negative values of *φ*. The corresponding structures are generally deformed counter-clockwise α-helixes. Analysis of such structures indicates that they represent important deviations from α and β forms: codes D and F are usually found at the ends of well-known secondary motifs (D for α-helixes and F for β-twists respectively). Termination of such motifs produces a new structural class (see Fig. 3.5 in [14] ) which is very important from the point of view of determining the overall conformation of larger residue chains. The greater predictive accuracy of the statistical dictionary method should be viewed as a significant advantage in this regard.

Another notable difference between the presented methods is the lower accuracy of the statistical dictionary method for cysteine residues (where only B-type structures are more accurately predicted than using the contingency table method). A decrease in accuracy is also observed for glycine (affecting 5 out of 7 structural codes), however the statistical dictionary method produces better results for G-type structures which are the most common conformation for this amino acid. The presented method is also less accurate with regard to B-type structures and—somewhat unexpectedly—C-type structures. Code C represents a clockwise helix which dominates the structure of many proteins. Results obtained using the older method suggest significant overrepresentation of helical fragments.

### Individual prediction examples

**Fig. 4:**
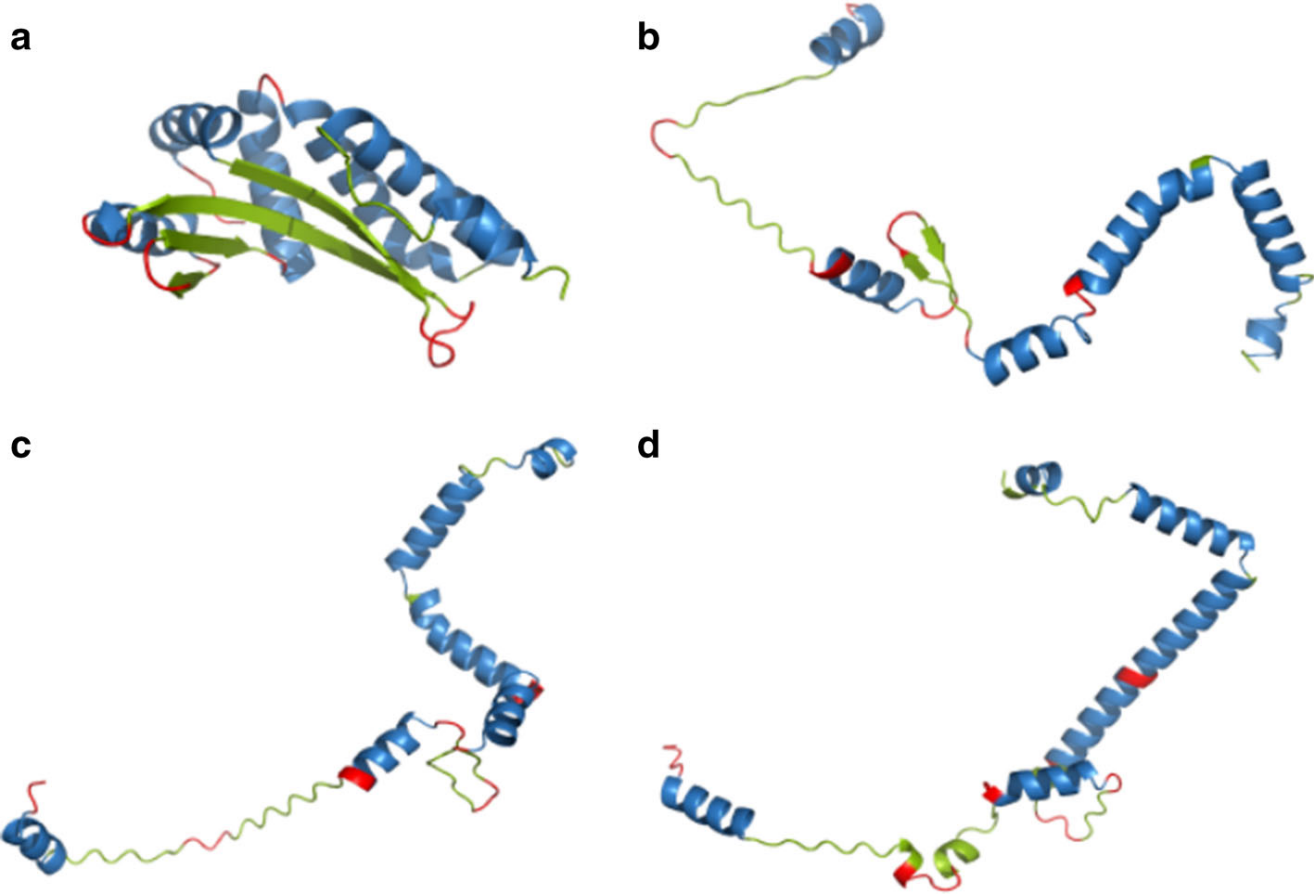
2VBL structure (A chain) **a** native structure derived from PDB, **b** structure obtained by projecting each (*φ*, *ψ*) angle pair onto the elliptical path which represents the ES conformational subspace, **c** ES structure obtained using the statistical dictionary method, **d** ES structure obtained using the contingency table method. *Blue*, *red* and *green* fragments correspond to residues which form α-helixes, β-twists and loops respectively. *Source*: PyMOL

For 2VBL the statistical dictionary method produced correct results in 92 % of cases. All α-helixes and β-twists were correctly predicted (Fig. 4), with incorrect structural codes occurring mainly at the ends of α-helixes. The contingency table (tetrapeptide) method achieved a much lower accuracy (51 %) with a marked overrepresentation of helical structures.

**Fig. 5:**
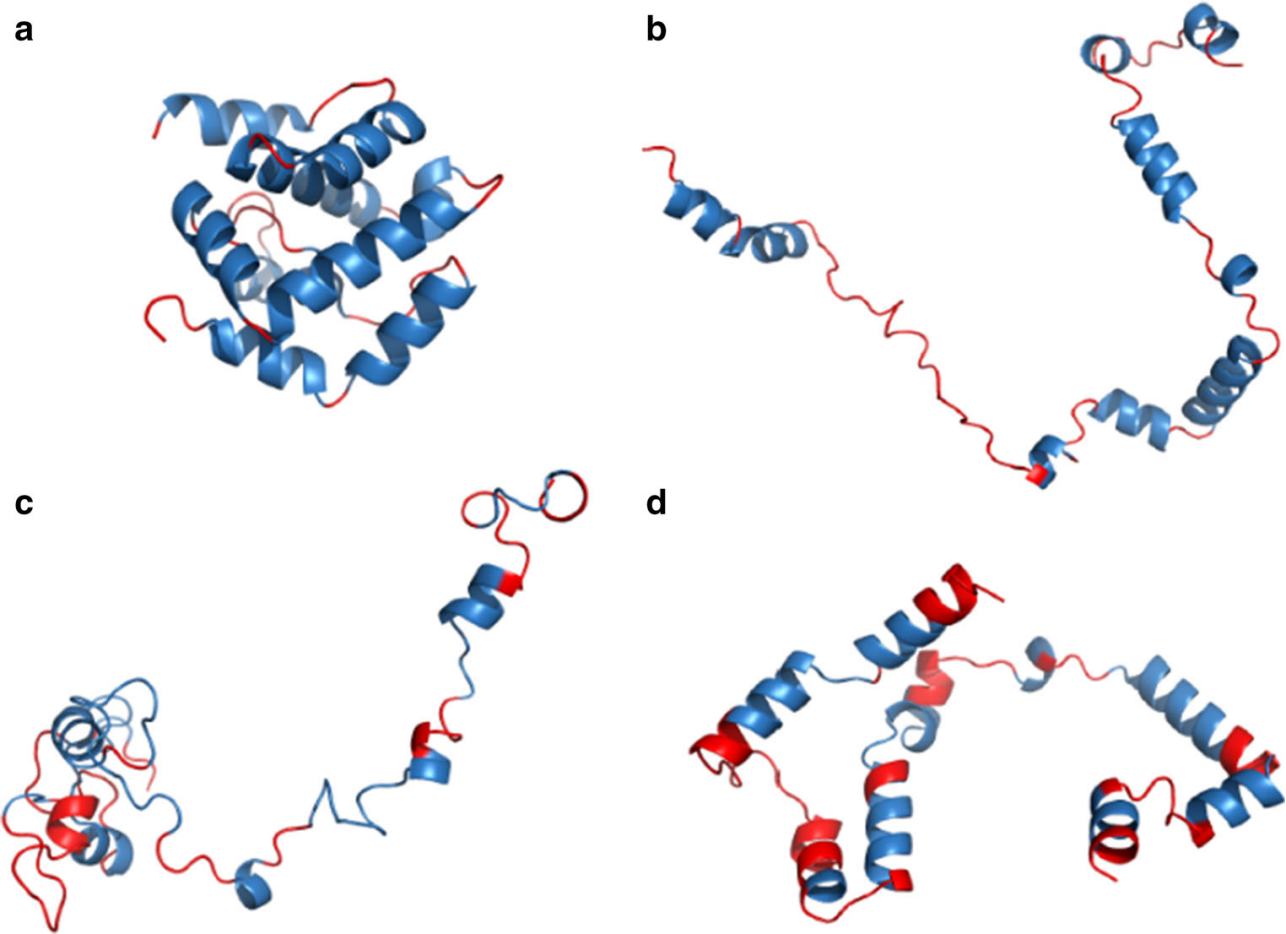
2JEK structure (A chain) **a** native structure derived from PDB, **b** structure obtained by projecting each (*φ*, *ψ*) angle pair onto the elliptical path which represents the ES conformational subspace, **c** ES structure obtained using the statistical dictionary method, **d** ES structure obtained using the contingency table method. *Blue*, *red* and *green* fragments correspond to residues which form α-helixes, β-twists and loops respectively. *Source*: PyMOL

2JEK is an example of a protein for which the statistical dictionary method produces less accurate results than the contingency table method (12 % decrease in accuracy). The statistical dictionary method is less apt to propose helical structures, which form the majority of this protein (Fig. 5).

**Fig. 6:**
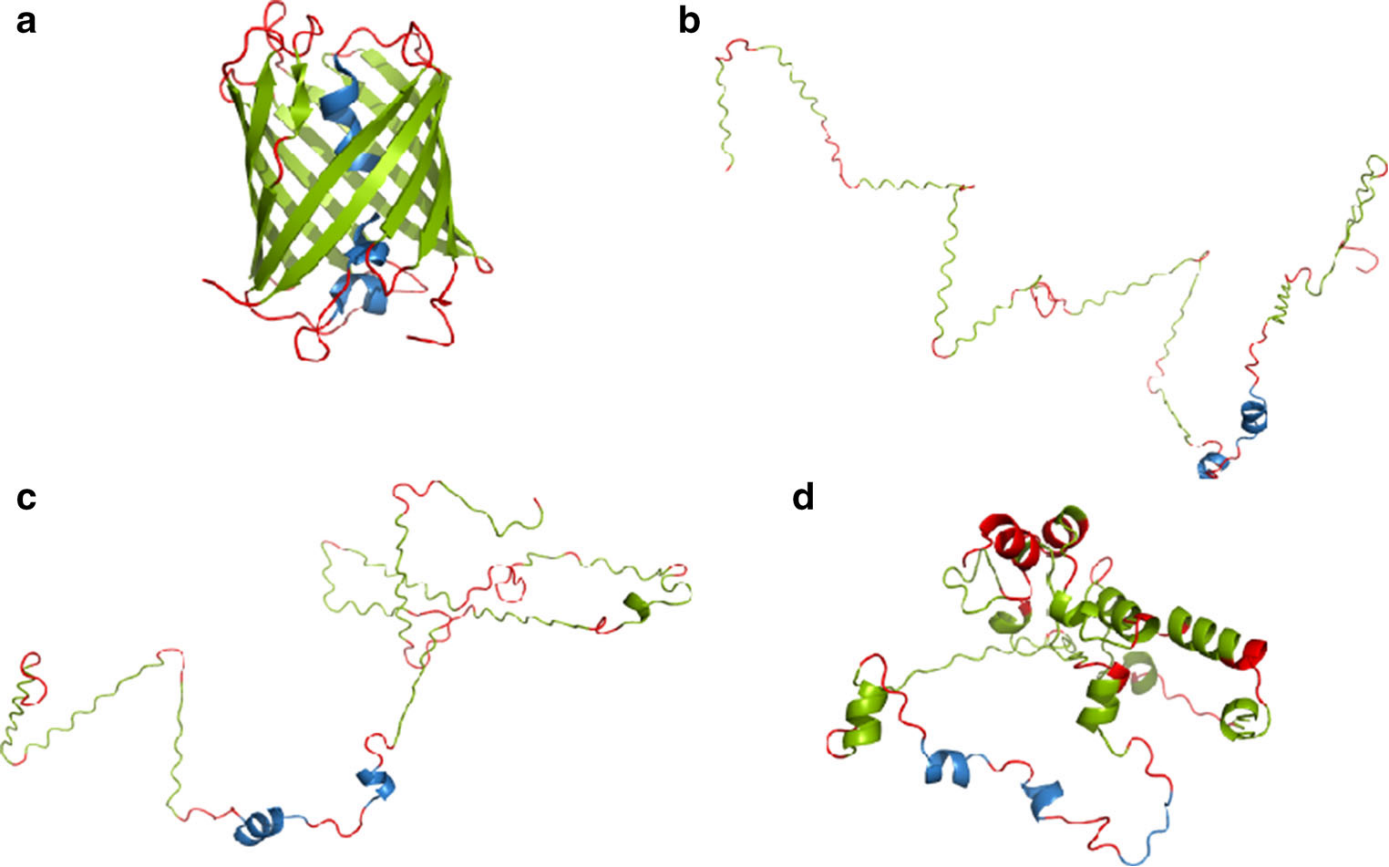
2VAD structure (A chain) **a** native structure derived from PDB, **b** structure obtained by projecting each (*φ*, *ψ*) angle pair onto the elliptical path which represents the ES conformational subspace, **c** ES structure obtained using the statistical dictionary method, **d** ES structure obtained using the contingency table method. *Blue*, *red* and *green* fragments correspond to residues which form α-helixes, β-twists and loops respectively. *Source*: PyMOL

The final example is 2VAD for which the statistical dictionary method proved vastly superior to the contingency table method (85 vs. 35 %). This particular protein consists mainly of β-sheets; a structural motif for which the contingency table method produces poor results. Figure 6 highlights the differences between the outcome of each algorithm, with extended fragments corresponding to individual β-sheets. Another possible reason for the reduced accuracy of the contingency table method is the potential presence of a ligand, which distorts the protein’s conformation.

Additional examples of structures predicted with particularly high or low accuracy are presented in Table 3.

**Table 3:** Best- and worst-case results using the statistical dictionary method. Comparative data obtained using the contingency table method for each structural code is given in parentheses [24]

PDB ID	Chain	Lenght	Accuracy (%) (previous results)	Main secondary structure	
1ICC	A	87	93.33 (51.25)	α and β	Best accordance
3CU4	A	85	93.15 (58.11)	α	
2VBL	A	153	92.37 (68.24)	α and β	
2H5U	A	499	91.68 (35.68)	β	
2R56	M	211	91.58 (47.03)	β	
2J04	D	109	32.82 (32.88)	β	Lowest accordance
2DMH	A	524	31.16 (38.41)	α and β	
1UEN	A	140	30.89 (32.48)	β	
2KCA	A	74	30.84 (37.74)	β	
1J3T	A	125	29.17 (33.82)	β	

Analysis of results listed in Table 3 confirms that the statistical dictionary method is less accurate when modeling helical structures. This is however, compensated for by its high accuracy with regards to β-twists and random coils (codes A, B, D and G), as confirmed by our analysis of 1CR9-L (immunoglobulin domain) and 1XAU-A (random coil).

### Comparison with SPINE X method

The accuracy of secondary structure prediction is presented in the Table 4. The level of correct prediction of helical structures is especially high for ES prediction method (78.3 %), while the SPINE-X method overpredicts coils (helixes—36.5 % and coils—48.4). The extended structures are predicted with similar accuracy by both methods. The SPINE-X allows users to predict coils with significantly higher accuracy (56.6 %, while only 11.5 % for the ES method). The ES prediction method does not distinguish turns and bends, which are included into coils class. The reason behind this is the location of many of such structures in C, E and F zones, what may cause high levels of prediction of these codes for the coils class.

**Table 4:** Prediction accuracy (in percentage) of the presented method and SPINE-X in relation to native secondary structures obtained by DSSP. In rows: percentage values of positions of a given DSSP class predicted as a structural class presented in columns

DSSP classes	ES structural codes’ groups (%)			SPINE-X secondary structural classes (%)		
	C	E, F	A, B, D, G	Helical	Extended	Coils
Helical	78.3	6.9	14.8	37.5	13.7	48.8
Extender	35.4	51.3	13.2	11.7	47.2	41.1
Coils	44.7	43.7	11.6	25.1	18.4	56.5

## Discussion and conclusions

In conclusion, it should be noted that the proposed method provides significantly more accurate results than the contingency table method [24] with an overall accuracy of 57 %. This accuracy seems sufficient given that determining the final structure of the target protein requires another simulation step—the late stage (LS) intermediate, which accounts for pair-wise interactions between atoms, as well as interactions between the polypeptide chain and its environment [13, 30, 31]. The main difficulty in modeling the ES intermediate lies in the lack of information regarding the molecule’s intended role—its biological specificity. The statistical dictionary method should be regarded as superior to the contingency table method as it acknowledges a broader neighborhood of each residue (compared to the tetrapeptide fragments, which form the basis of the contingency tables). This results in better prediction accuracy, particularly in the scope of D and F motifs which correspond to the terminal parts of α-helixes and β-twists respectively. Of note is the reduced accuracy in predicting cysteine and glycine conformations—this, however, can be alleviated by incorporating elements of the contingency table analysis algorithm into the proposed method. The further work assumes the analysis of non-redundant data base with <30 % sequence similarity. The comparative analysis of these two data base may deliver information about possible influence of homology sequence on the final prediction.

The detailed analysis of (*φ*, *ψ*) angles distribution additionally suggests the possible incorporation of the zone B to the zones E and/or F. Elimination of B of low probability observed for this zone may significantly improve the prediction reliability of the model. The discussion of the effect of ligand binding seems unrelated to the model under consideration. However the late stage model taking in consideration the interaction of folding polypeptide with the surrounding environment (water and ligands) seems to be significantly sensitive to the external molecules. This was the reason to distinguish the status of particular residue in respect to possible interaction influencing its conformation. The comparative analysis (Table 4) reveals much better prediction of random coil structures SPINE-X, however the others recognitions seem to be of similar efficiency.

Besides the methods based on theoretical calculations some experiments deliver valuable information about the ES steps of protein folding process. Experimental observations [for example hydrogen-exchange pulse-labelling mass-spectrometry method applied for large two-domain maltose binding protein (MBP; 370 residues)] suggest the presence of intermediate composed of segments that are distant which generate the immediate interaction and final collapse in the next steps of folding process [32]. However ab inito methods are limited to the proteins of domain-like size pf about 100–120 aa. This is why the experimental analysis of small molecules like RNase H (152 aa 1F21) may the perfect object for verification of theoretical methods simulating folding process and protein structure prediction [33].

## References

[1] Bystroff C, Shao Y, Bujnicki J (2004). Modeling protein folding pathways. Practical bioinformatics.

[2] Feng H, Zhou Z, Bai Y (2005). A protein folding pathway with multiple folding intermediates at atomic resolution. Proc Natl Acad Sci USA.

[3] Galzitskaya OV, Ivankov DN, Finkelstein AV (2001). Folding nuclei in proteins. FEBS Lett.

[4] Grantcharova VP, Baker D (1997). Folding dynamics of the src SH3 domain. Biochemistry.

[5] Jha SK, Marqusee S (2014). Kinetic evidence for a two-stage mechanism of protein denaturation by guanidinium chloride. Proc Natl Acad Sci USA.

[6] Kuwajima K, Schmid FX (1984). Experimental studies of folding kinetics and structural dynamics of small proteins. Adv Biophys.

[7] Religa TL, Markson JS, Mayor U, Freund SM, Fersht AR (2005). Solution structure of a protein denatured state and folding intermediate. Nature.

[8] Yamada S, Ford NDB, Keller GE, Ford WC, Gray HB, Winkler JR (2013). Snapshots of a protein folding intermediate. Proc Natl Acad Sci USA.

[9] Duan Y, Kollman PA (1998). Pathways to a protein folding intermediate observed in a 1-microsecond simulation in aqueous solution. Science.

[10] Huang L, Shakhnovich EI (2012). Is there an en route folding intermediate for cold shock proteins?. Protein Sci.

[11] Alejster P, Jurkowski W, Roterman I, Roterman-Konieczna I (2012). Structural information involved in the interpretation of the step-wise protein folding process. Protein folding in Silico.

[12] Roterman I, Konieczny L, Banach M, Marchewka D, Kalinowska B, Baster Z, Piwowar M, Liwo A (2013). Simulation of protein folding process. Computational methods to study the structure and dynamics of biomolecules and biomolecular processes.

[13] Roterman I, Konieczny L, Banach M, Jurkowski W (2011). Intermediates in the protein folding process: a computational model. Int J Mol Sci.

[14] Roterman I, Konieczny L, Jurkowski W, Prymula K, Banach M (2011). Two-intermediate model to characterize the structure of fast-folding proteins. J Theor Biol.

[15] Brylinski M, Jurkowski W, Konieczny L, Roterman I (2004). Limitation of conformational space for proteins—early stage folding simulation of human α and β hemoglobin chains. TASK Q: Sci Bull Acad Comput Cent Gdansk.

[16] Bryliński M, Jurkowski W, Konieczny L, Roterman I (2004). Limited conformational space for early stage protein folding simulation. Bioinformatics.

[17] Bryliński M, Konieczny L, Czerwonko P, Jurkowski W, Roterman I (2005). Early-stage folding In proteins (in silico)—sequence-to-structure relation. J Biomed Biotechnol.

[18] Jurkowski W, Baster Z, Dułak D, Roterman I, Roterman-Konieczna I (2012). The early stage intermediate. Protein folding in Silico.

[19] Jurkowski W, Brylinski M, Konieczny L, Roterman I (2004). Lysozyme folded in silico according to the limited conformational sub-space. J Biomol Struct Dyn.

[20] Jurkowski W, Brylinski M, Konieczny L, Wiśniowski Z, Roterman I (2004). Conformational subspace in simulation of early-stage protein folding. Proteins.

[21] Jurkowski W, Kułaga T, Roterman I (2011). Geometric parameters defining the structure of proteins relation to early-stage folding step. J Biomol Struct Dyn.

[22] Roterman I (1995). Modelling the optimal simulation path in the peptide chain folding—studies based on geometry of alanine heptapeptide. J Theor Biol.

[23] Alonso DO, Daggett V (1998). Molecular dynamics simulations of hydrophobic collapse of ubiquitin. Protein Sci.

[24] Kalinowska B, Alejster P, Sałapa K, Baster Z, Roterman I (2013). Hypothetical in silico model of the early-stage intermediate in protein folding. J Mol Model.

[25] Cormen TH, Leiserson CE, Rivest RL, Stein C (1990). Introduction to algorithms.

[26] Joosten RP, Beek TAH, Krieger E, Hekkelman ML, Hooft RWW, Schneider R, Vriend G (2011). A series of PDB related databases for everyday needs. Nucleic Acids Res.

[27] Kabsch W, Sander C (1983). Dictionary of protein secondary structure: pattern recognition of hydrogen-bonded and geometrical features. Biopolymers.

[28] Faraggi E, Xue B, Zhou Y (2009). Improving the prediction accuracy of residue solvent accessibility and real-value backbone torsion angles of proteins by fast guided-learning through a two-layer neural network. Proteins.

[29] Faraggi E, Yang Y, Zhang S, Zhou Y (2009). Predicting continuous local structure and the effect of its substitution for secondary structure in fragment-free protein structure prediction. Structure.

[30] Banach M, Prymula K, Jurkowski W, Konieczny L, Roterman I (2012). Fuzzy oil drop model to interpret the structure of antifreeze proteins and their mutants. J Mol Model.

[31] Sarkar SS, Udgaonkar JB, Krishnamoorthy G (2013). Unfolding of a small protein proceeds via dry and wet globules and a solvated transition state. Biophys J.

[32] Walters BT, Mayne L, Hinshaw JR, Sosnick TR, Englander SW (2013). Folding of a large protein at high structural resolution. Proc Natl Acad Sci USA.

[33] Hu W, Walters BT, Kan ZY, Mayne L, Rosen LE, Marqusee S, Englander SW (2013). Stepwise protein folding at near amino acid resolution by hydrogen exchange and mass spectrometry. Proc Natl Acad Sci USA.

